# In platelet single donor apheresis, platelet factor 4 levels correlated with donor’s age and decreased during storage

**DOI:** 10.1038/s41598-024-56826-4

**Published:** 2024-03-14

**Authors:** Anne Claire Duchez, Marco Heestermans, Charles-Antoine Arthaud, Marie-Ange Eyraud, Mailys Portier, Amélie Prier, Hind Hamzeh-Cognasse, Fabrice Cognasse

**Affiliations:** 1 INSERM, U 1059 SAINBIOSE, Université Jean Monnet, Mines Saint-Étienne, 42023 Saint-Etienne, France; 2 Establissement Français du Sang Auvergne-Rhône-Alpes and INSERM U1059, 25 Boulevard Pasteur, 42100 Saint-Etienne, France

**Keywords:** Immunology, Biomarkers, Medical research

## Abstract

The human population is ageing worldwide. The World Health Organization estimated that the world’s population of people aged 60 years and older will increase to at least 30%, coinciding with a growing frequency of cognitive and cardiovascular disease. Recently, in preclinical studies platelet Factor 4 (PF4) was presented as a pro-cognitive factor. This molecule is released by platelets in the circulation and could be present in blood products destined for transfusion. We wondered if PF4 levels are correlated to the age of the blood donor or to the storage time of platelet concentrates (PCs) intended for transfusion? We observed higher levels of PF4 in PCs from elderly donors compared to younger donors, while PC storage time did not determine PF4 levels expression.

On 15 June 1667, Dr Jenys, assisted by Dr Emmerez performed the first fully documented blood transfusion. Approximately twelve ounces of lamb's blood were transfused into the veins of a 15-year-old boy who had been suffering from uncontrollable fever for two months. Interestingly, one year later, a dementia patient was also given a blood transfusion by Denys and Emmerez. This marked the advent of a putative link between blood transfusion and cognitive pathologies—currently a forgotten “connection”.

More recently, studies making use of heterochronic parabiosis connecting old and young mice have demonstrated systemic rejuvenating properties^1,2^. In addition, blood plasma transfer from young to older mice reversed age-related cognitive impairments, although the exact mechanisms involved are unclear^1–5^. A few molecules however have been placed in the limelight to be involved in this proces. Eotaxin-1 or TIMP-2 detected in blood components prepared for transfusion have been associated with cognitive function and ageing^6^, as well as Platelet Factor 4 (PF4), which is mainly released from platelet α-granules^7^.

Platelets are anucleate cells and are well known for their haemostatic function, but less for their immune function. Platelets are mostly transfused as platelet concentrates (PCs), generated from full blood donations. Importantly, they are potent secretors of numerous soluble factors, such as prostaglandins and cytokines including PF4^7^.

PF4 was one of the first cytokines to be discovered (first as chemokine ligand 4) and has been highly conserved during evolution^8,9^. The main physiological function of PF4 is the promotion of blood haemostasis but it also mediates the immune response. During coagulation and clot formation, PF4 interact with the endothelium, monocytes and neutrophils and participate in the generation of Neutrophil Extracellular Traps^10,11^. In addition, PF4 binds to bacteria^12^. The host can subsequently generate auto-antibodies against PF4, mediating the opsonisation of bacteria and FCγRIIa on platelets, which are able to kill bacteria^12,13^. Because of the ability of PF4 to generate auto-antibodies it is also a major player in heparin induced thrombocytopenia and vaccine-induced immune thrombotic thrombocytopenia^14–18^.

Recently published reports propose a new role for PF4 to be involved in cognitive disorders^3–5^. Indeed, PF4 administration decrease hippocampal neuroinflammation and improved cognition in aged mice^3^. Administration of Klotho, a cognition-enhancing protein, increases PF4 levels. PF4 permeates the brain and enhances cognition in mice^4^. PF4 is released by physical-activity-inducing-activated platelet, and PF4 induces proliferation of hippocampal precursor cell, leading to cognitive enhancement^5^.

A significant proportion of patients who undergo major surgery experience temporary or permanent decline in cognitive performance: Post-Operative Cognitive Dysfunction (POCD)^19^. These patients received mainly platelet concentrate (PC) transfusion. During collection, preparation and storage, PCs destined for transfusion experience stress-induced lesions^20^. Platelet processing may alter structure and function, such as an increased Biological Response Modifier (BRM) secretion. These BRMs range from cytokines to extracellular vesicles, lipids and soluble Danger-Associated Molecular Patterns^21–23^. Not a lot is known of PF4 modulation during PC storage.

We therefore wondered whether donor age influences the levels of “rejuvenating” PF4 and whether PF4 levels alter during storage of PCs. Our study investigated the effects of donor age and single donor apheresis platelet concentrate (SDA-PC) storage time on PF4 levels.

## Results

### Blood donor characteristics

Recent reports demonstrated that PF4 is involved in systemic inflammation and cognitive repair^1,3–5^. These observations made us wonder whether SDA-PC level of PF4 are linked to donor age and/or storage time. We obtained residual fractions of transfused SDA-PCs, from which platelets were discarded to keep only the supernatant for PF4 analysis (Supplementary Fig. 1a). We analysed in total 1,707 SDA-PCs (1,092 males and 615 females) from donors aged 18 to 65 years (Supplementary Fig. 1b). The SDA-PCs were also subdivided into three categories based on storage time: 0, [1–3], and [3–5] days. Stored SDA-PCs could be transfused until study day 5 at the time of the study after which they are discarded. The majority of donors were male aged 30 to 60 (Supplementary Fig. 1c). The same pattern was observed with different storage times (Supplementary Fig. 1c).

### PF4 levels associated with increasing age

**Figure 1:**
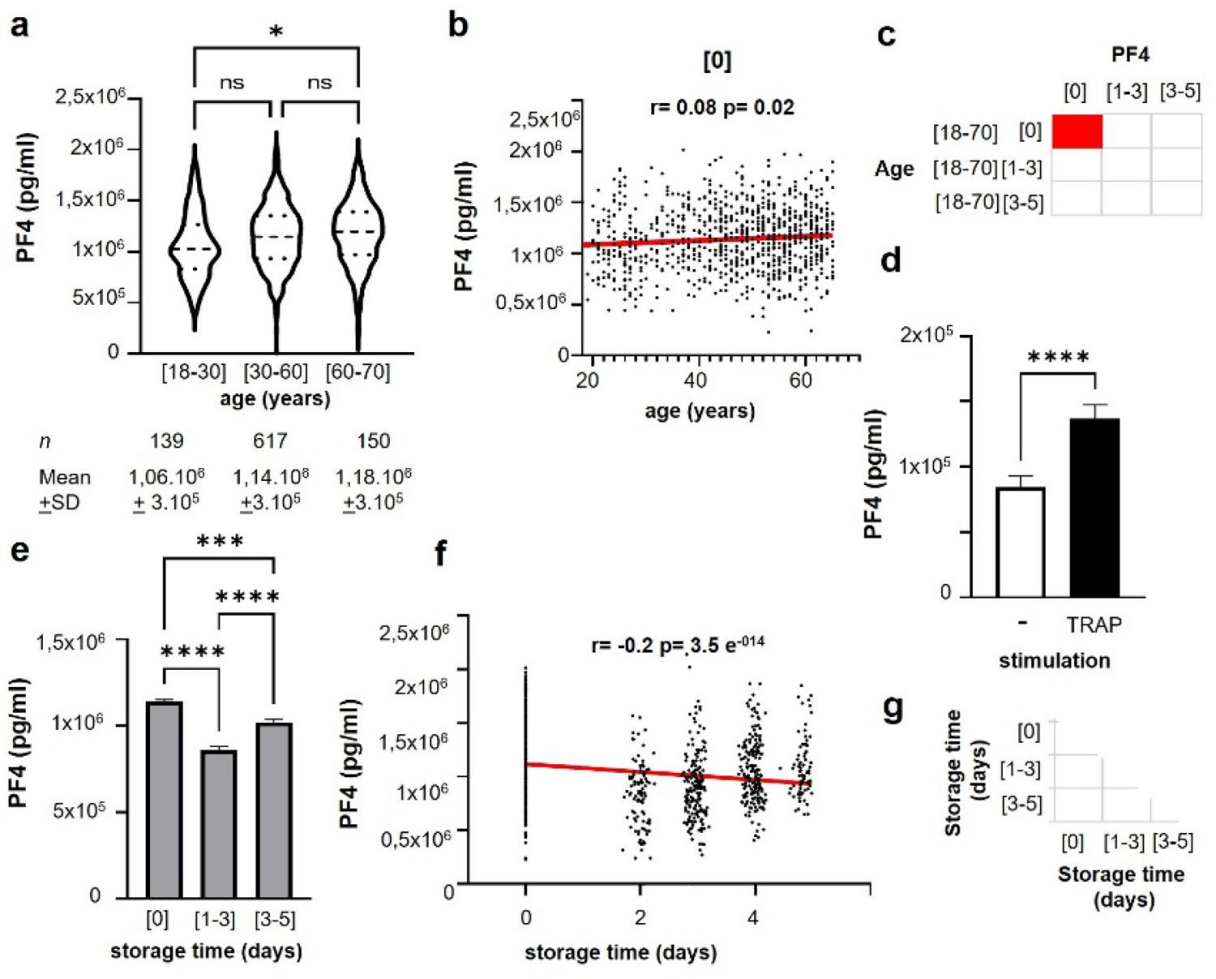
Evaluation of SDA-PC PF4 levels depending on donor age and storage time independently. (**a**) Velocity plot of the expression of SDA-PC PF4 based on different donor age, 2-way ANOVA, *p < 0.05; non-significant (ns); large dash present median of data and small dash the quartiles. (**b**) Graph illustrating PF4 expression in function of donor age at day [0] of storage time. Regression line in red, spearman correlation r = 0.08; *p* value = 0.02 *. (**c**) Spearman’s correlation matrix between PF4 concentration and age, based on storage time. Red square corresponds to positive correlation with *p* value < 0.05; white square corresponds to no correlation with *p* value < 0.05. (**d**) Graph bar representing the release of PF4 after a stimulation with TRAP platelet agonist in Platelet Rich Plasma (PRP); n = 21; Wilcoxon test *p* < 0.0001 **** (**e**) PF4 expression during storage period regardless of donor age, 2-way ANOVA, ***p < 0.001 ****p < 0.0001. (**f**) Graph illustrating PF4 expression in function of storage time independently of age donor. Regression line in red, spearman correlation r = 0.31 *p* value = 1.32 e^-13^ *. (**g**) Spearman’s correlation matrix between PF4 concentration and storage time. White square corresponds to no correlation with p value < 0.05.

During the first analysis, PF4 levels from SDA-PCs were plotted according to the donor’s age, regardless of PC storage time (Fig. 1a). PF4 was significantly elevated in elderly donors (over 60), as compared to younger donors (Fig. 1a). At storage time [0], PF4 levels correlated positively with donor age (Fig. 1b,c). No statistically significant correlation was observed between donor’s age and PF4 expression at storage time [1–3] and [3–5] (Fig. 1c). It is in line with an increase of platelet’s activation when Platelet Rich Plasma is stimulated with the platelet agonist thrombin receptor activator peptide (TRAP). We observed a significant increase of PF4 levels in the supernatant of PRP stimulated with TRAP (136.9 ng/ml + 10.5), as compared to unstimulated PRP (84.3 + 8.5 ng/ml) (Fig. 1d).

Moreover, PF4 expression did not seem to be linked to sex of the donor, since we did not observe any significant differences of PF4 level in male or female donors in all different age categories (Supplemental Fig. 2a,b).

**Figure 2:**
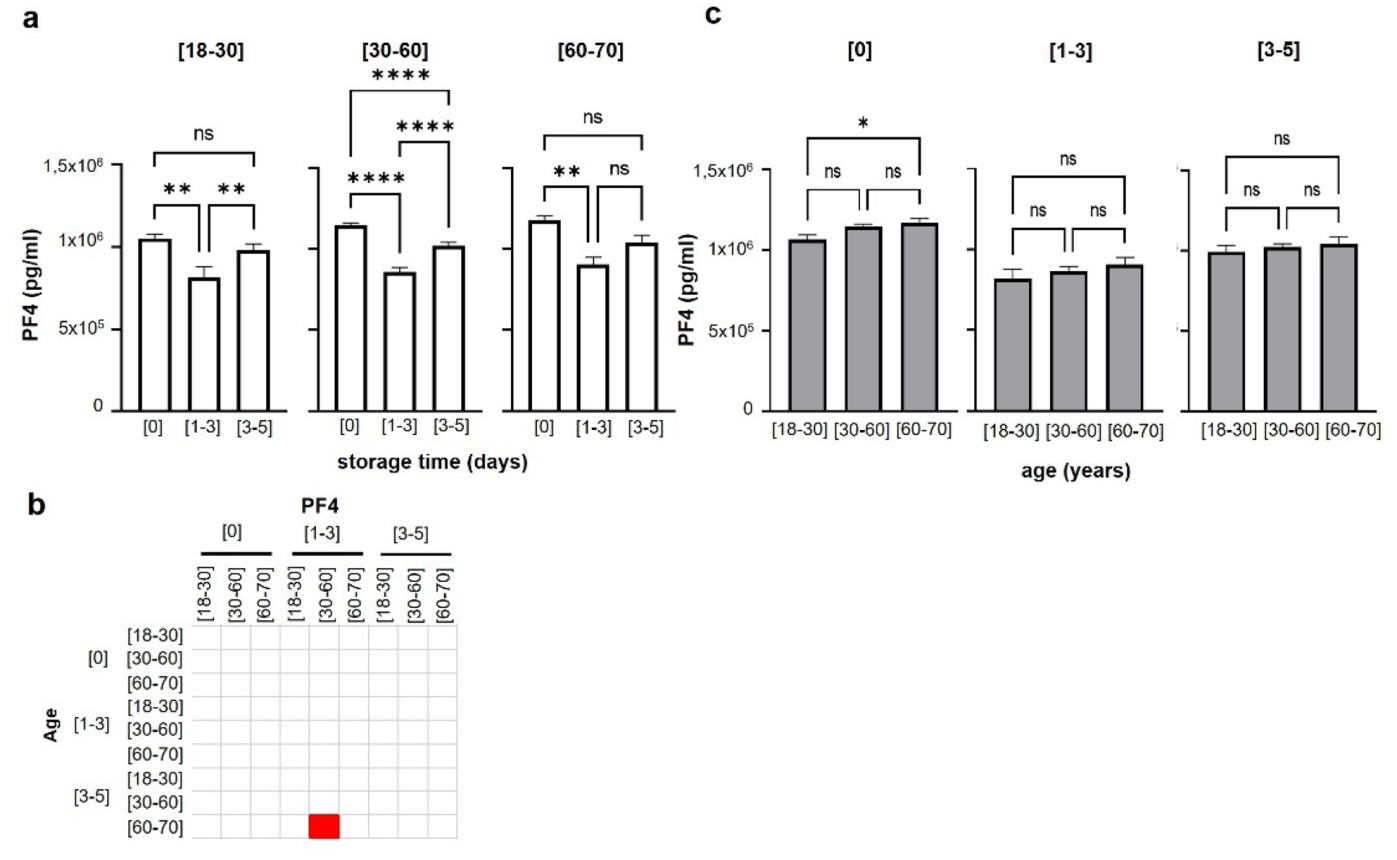
Evaluation of SDA-PC PF4 levels depending on donor’s age and storage time. (**a**) PF4 expression based on donor age and storage time 2-way ANOVA, **p < 0.01, ****p < 0.0001, non-significant difference (ns). (**b**) Graph bar represents PF4 expression based on storage time ([0], [1–3] and [3–5] days, 2-way ANOVA, *p < 0.05 (**c**) Spearman’s correlation matrix based on different donor age groups, storage time and PF4 concentrations. Red square corresponds to positive correlation with *p* value < 0.05; white square corresponds to no correlation with *p* value < 0.05.

### PF4 levels correlated with storage time

We wondered whether PF4 levels could be modulated by the storage of SDA-PCs, regardless of donor age. Interestingly, storage appeared to significantly modulate, decreasing PF4 levels (Fig. 1e). No significant correlations were detected by spearman correlation between sample stored between [0–5] days and PF4 level (Fig. 1f,g).

When dividing the SDA-PCs in different donor age groups followed by a subanalysis on storage time, we noted that PF4 levels were significantly higher at storage time [0] compared to [1–3] days of storage, for all donor ages. We noticed a significant decrease of PF4 levels between [0] and [1–3], in donors of [18–30], [30–60] and [60–70] years old (Fig. 2a). Furthermore, a slight increase in PF4 expression was, however, noted between days [1–3] and [3–5] only for donors between [18–30] and [30–60] years (Fig. 2a). The same pattern was observed with [60–70] year old donors, but not significant (Fig. 2a). Interestingly, PF4 levels did not differ for donors aged [18–30] and [60–70] between [0] to [3–5] days of storage, while they did significantly differ for donors aged [30–60] (Fig. 2a). No significative correlation was noticed between [0] and [3–5] storage time in [18–30] and [60–70] aged donors (Fig. 2b).

We compared the influence of storage time on PF4 levels in different age groups (Fig. 2c). We also analysed the storage time data set plotted with donor age (Fig. 2c). No significant change in PF4 levels over storage times [1–3] and [3–5] were observed with the different donor ages (Fig. 2c). However, a significant increase in PF4 levels between younger (18–30) and older (60–70) donors was observed at storage time [0] (Fig. 2a). Finally, there is no significant, positive correlation between PF4 and storage time and donor age (Fig. 2b).

**Figure 3:**
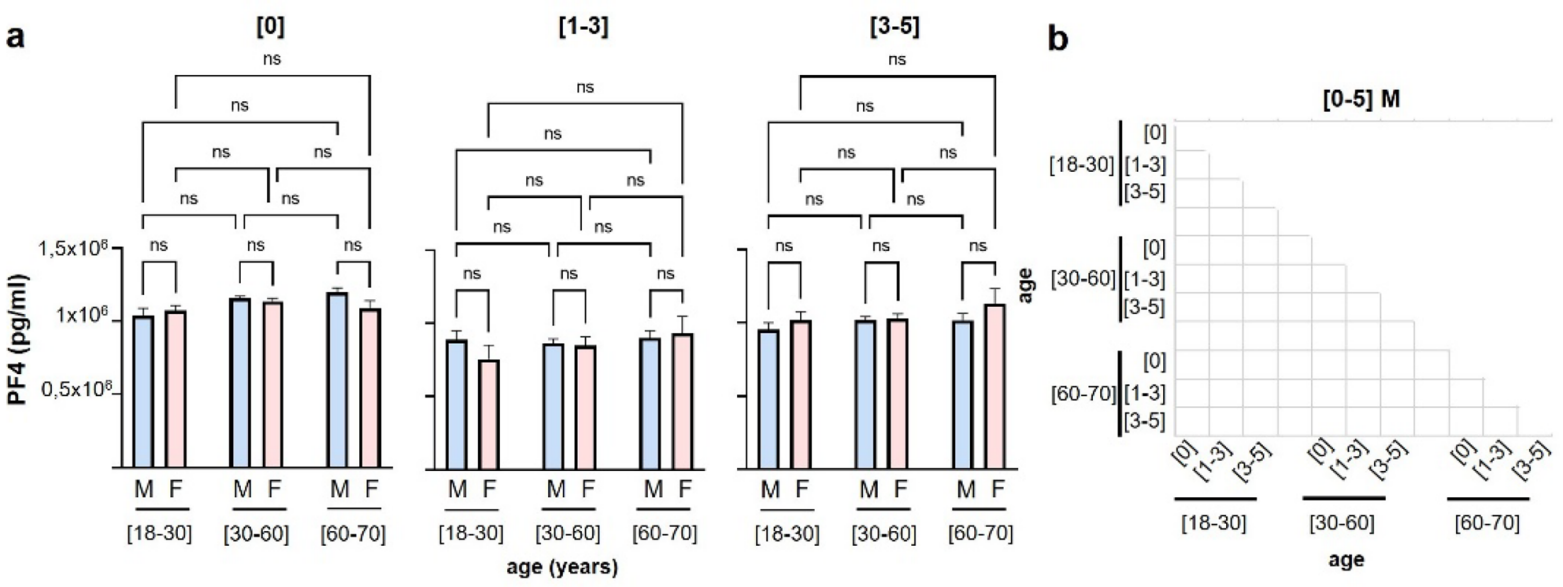
Evaluation of SDA-PC PF4 levels depending on donor’s age, storage time and sex. (**a**) Graph bar represents PF4 expression based on storage time ([0], [1–3] and [3–5] days, 2-way ANOVA. Light Blue for male donors (M) and light pink for female donors (F). (**b**) Spearman’s correlation matrix based on different donor age groups, storage time and PF4 concentrations. White square corresponds to no correlation with *p* value < 0.05.

PF4 expression was neither modulated by sex of the donor (Supplemental Fig. 2), nor was there a correlation between storage and sex (Fig. 3). There is no significant difference between PF4 levels in male and female donors during the storage of the SDA-PC (Fig. 3a), neither a significant correlation between age and storage in male donors (Fig. 3b).

## Discussion

Recent reports demonstrated a lower expression of PF4 in elderly human donors and mice^3,24^. Remarkably, the exogenous administration of PF4 appeared to be beneficial for the restoration of cognitive function in aged mice, hinting towards a novel revolutionary therapeutic strategies to combat cognitive disorders^5^. However, our data showed quite the opposite. We measured higher PF4 levels in supernatants of SDA-PCs from elderly donors compared to younger donors, but we did not evaluate cognitive function of the donors.

Among different studies, the normal value of PF4 was 6.6 ng/ml (median) with 90% confidence interval between 1.7 ng/ml and 20.9 ng/ml^25^, 7.4 ng/ml (90% confidence interval 4 to 24)^26^, or 102 ng/ml + 32 (range 50–160) in plasma^27^. In our study, we have almost a factor 10 to 100 increase, since we measured PF4 in the supernatant of SDA-PC, which is a concentrate of platelets thus logically containing higher levels of platelet-secreted factors. In blood, the range for human platelets is around 120,000 to 450,000 platelets/mL, whereas the platelet concentration in SDA-PCs is around 1,500,000 platelets per mL. In the study from Schroer et al., the authors detected around 75 ng/mL of PF4 in young male mice. Over the time course of 24 h, they injected PF4 8 times in a concentration of 5 µg/mL to detect changes in inflammation and cognitive function in mice^3^. Schroer et al. perform their study only on male human blood donors, while the age groups were similar to the ones from our study. They categorized however the young group from [22–34] years old and the elderly as [61 to 75] years old^3^. In our study, our elderly group is under 65 years old, younger donor as studied by Schroer et al., maybe it can explain the difference in level of PF4. Moreover, PCs could contain activated platelet and the increase in PF4 level is a reflection of platelet activation in PCs. We also mixed genders during the first analysis, after which we also performed distinguishing PF4 level data by sex of donor (Supplemental Figs. 2 and 3). We did not notice any modulations of PF4 based on gender.

**Figure 4:**
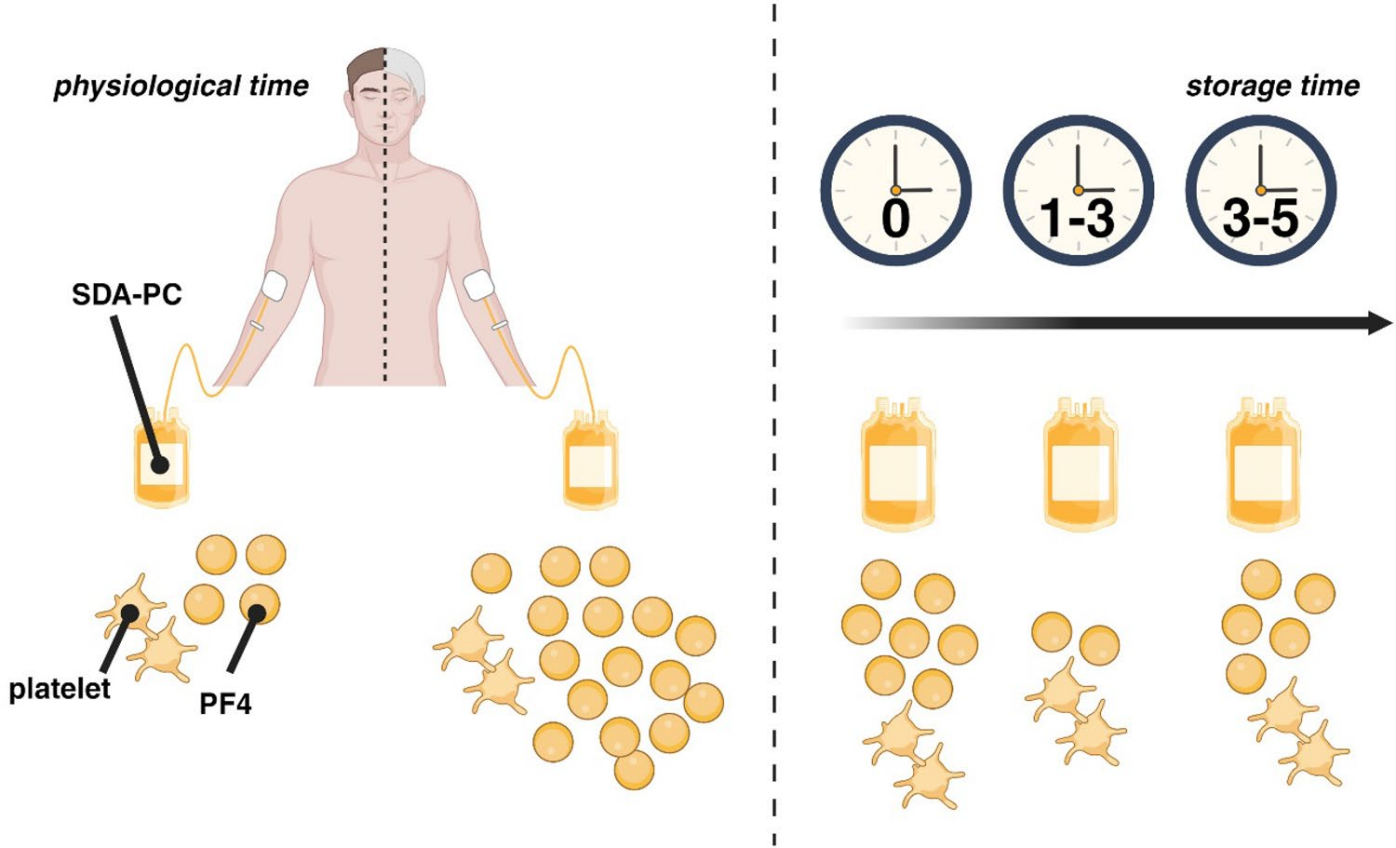
Summary of the study. Left panel. Our study compared the level of platelet factor 4 (PF4), mainly produced/stored by platelet, in single donor apheresis platelet concentrate (SDA-PC), based on the age of the donor (physiological time). Right panel. Our study, also, compared the PF4 level during the storage time of SDA-PC. We conclude that elderly donors have a higher concentration of PF4 in SDA-PCs, compared to younger donors (left panel). We also conclude that PF4 level is modulated through the storage time with no difference between the beginning [0] days and the end of the storage [3–5] days (right panel).

PF4 levels increase slightly during storage time of the SDA-PCs (Fig. 4).

Storage time of SDA-PCs (from day [0] to [3–5]) may impact PF4 levels, depending on donor age (Fig. 2a). We observed a slight but significant decrease of PF4 levels between [0] and [1–3] days of storage, which could be explained by PF4 consumption by or binding to platelets, via receptors such as CXCR3, LRP/LDLR, or proteoglycans^8,9^.

An initial glance at SDA-PC storage did not highlight any significant changes in PF4 levels (Fig. 2c). Based on our data and the work of Leiter et al*.*^5^, new transfusion approaches could be explored. The scientific community and blood bank worldwide should study the impact of PCs from elderly donors or shortly stored (both rich in PF4) in their transfusion protocols, which could serve as a an add-on running therapies for patients with cognitive disease. Considering that PF4 is proposed as a pro-cognitive factor with potentially beneficial therapeutic applications in age-related neurodegenerative diseases^1,3–5^, PF4-enriched-PCs could provide beneficial therapeutic transfusions in age-related neurodegenerative disease. A possible PF4 spike in SDA-PCs during preparation and storage could improve the potential of PC transfusion in age-related neurodegenerative diseases. This hypothesis needs to be further studied, as the world population is ageing which coincides with an increase of neurodegenerative and cognitive diseases.

Our study aligns with numerous works related to personalized transfusions, which is based on transfusing the best blood products tailored to the individual patient^28^. This approach will still require however extensive research, to equip physicians in transfusion medicine with a range of novel toolsets. This will enable the reevaluation of the interplay between donor and recipient characteristics, thereby enhancing the significance of personalized transfusion medicine.

Since JB Denys and P. Emmerez in the seventeenth century, the advent of a putative link between blood transfusion and cognitive pathologies—a forgotten “connection”—is still open for investigation. Heterochronic parabiosis can connect an elderly mouse to a younger mouse, after which the biological fluids such as blood are shared. Using this technic, several studies pointed out that it is possible to commence a rejuvenating process, such as a reprogramming of the transcriptome by inhibiting their ageing signature^2,29,30^. The immune system is also affected by the heterochronic parabiosis^30,31^. Indeed, younger mice in the parabiosis system are not able to “rejuvenate” the immune system of the elderlies mice^31^. In line with observations from Denys & Emmerez and the literature on parabiosis, the scientific community needs to test transfusions on older mice with a PF4 rich product and evaluate the rejuvenation of the mice. Combined with other studies, PF4 is a promising molecule possibly crucial for neurodegenerative and cognitive diseases, and could be used both as a biomarker and as a therapeutic.

## Methods

### Ethics and blood donors

This study complies with all relevant ethical regulations. Fresh blood from healthy donors was obtained from Etablissement Français du Sang (EFS) upon approval of the project by Ministry of Higher Education and Research (authorization number CODECOH DC-2019-3803 & AC-2020-3959). The study was approved by the national review board for biomedical research in March 2017 (Comité de Protection des Personnes Sud-Est I, Saint-Etienne -, France; ID RCB Number: 2014- A00405-42), in agreement with the General Data Protection Regulation (Regulation (EU) 2016/679 and Directive 95/46/EC) and the French data protection law (Law n°78-17 on 06/01/1978 and Décret n°2019-536 on 29/05/2019). Informed consent was obtained from all subjects and/or their legal guardian(s). Our study involves 1,707 donors (1,092 males and 615 females) between 18 and 70 years of age (Supplemental Fig. 1).

### Blood preparation

As described previously^32^, single-donor apheresis (SDA) PCs were collected from donors and processed at the EFS. Briefly, SDA-PCs were leuko-reduced to < 10^6^/bag and suspended in 35% native plasma/65% nutritive solution (InterSol™(Fenwal, Lake Zurich, IL, USA), called PAS-C/PAS-III), or SSP^+^™ (Macopharma, Tourcoing, France), called modified PAS-E/PAS-IIIM. SDA-PCs were kept at 22 + 2 °C under shaking (60 rpm) until they were transfused. At the time of the study, SDA-PCs were transfused until 5 days post-collection, after which they were destroyed.

For the study, SDA-PCs stored 0 to 5 days were used for transfusion. The left-over of transfusion was collected and platelets contained in SDA-PCs were discarded after centrifugation (450 g, 10 min), after which the supernatants were stored at -80 °C until PF4 measurement^32^. The supernatants of SDA-PC were quickly thawed before the PF4 measurement by ELISA (Supplemental Fig. 1a).

### Platelet Rich Plasma preparation

Platelet-rich plasma (PRP) was prepared as previously described^33^. Briefly, peripheral blood was collected from healthy donors in endotoxin-free tubes with 3.2% sodium citrate (Becton Dickinson, Franklin Lakes, New Jersey, USA) and centrifuged at 150 g for 10 min at 22 °C. PRP was stimulated for 30 min at room temperature with 50 µg/mL thrombin receptor activator peptide (TRAP-SFLLRN, Sigma-Aldrich, Burlington, Massachusetts, USA). After stimulation, PRP was centrifuged at 2500 g 10 min, and the supernatants were collected and kept frozen at −80 °C until further analysis.

### ELISA PF4

ELISA kit (PF4 DuoSet, ref. DY795—R&D Systems, Minnesota, USA) was used to measure PF4 levels, following the manufacturer’s instructions. Absorbance at 450 nm was determined with an ELISA plate reader (Magellan Sunrise, Tecan Group Ltd., Mannedorf, Switzerland).

### Statistical analysis

The statistical analysis was carried out using GraphPad version 9 (La Jolla, CA). Normality of the data set was analysed through Kolmogorov–Smirnov test. Our data sets were not normally distributed. Comparison between sets of data was performed with a 2-ways ANOVA test, with Tukey multiple comparisons correction. Correlation matrix was performed with a spearman correlation.

### Biorender

The cartoon at Fig. 3 was made via Biorender, Agreement numbers BA266PYIBY and UB26G9VXSY.

### Supplementary Information

Supplementary Information.

## Data Availability

The datasets used and/or analysed during the current study available from the corresponding author on reasonable request.
