# Supplementary material for: In platelet single donor apheresis, platelet factor 4 levels correlated with donor’s age and decreased during storage
Source: Sci Rep. 2024 Mar 14;14:6231. doi: 10.1038/s41598-024-56826-4 (PMC10940288; doi:10.1038/s41598-024-56826-4)
Supplement: Supplementary file 1 — Supplementary Information. [file 41598_2024_56826_MOESM1_ESM.pdf]

## Supplementary files

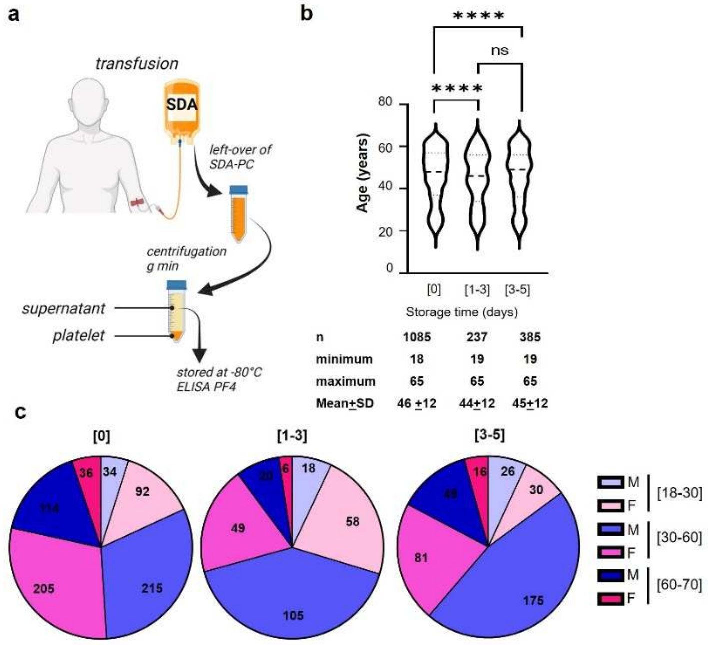


**<u>Supplemental Figure 1.</u> Donor profiles.** (a) Schematic overview representing the processing of SDA-PCs in our study. (b) Violin donor age plot of PF4 levels, depending on SDA-PC storage time, 2-way ANOVA, **** $p < 0.0001$. The quartiles are represented with small dashes and medians with large dash. (c) Pie chart illustrating donor distribution, according to sex and age. Male donor (M); female donor (F)

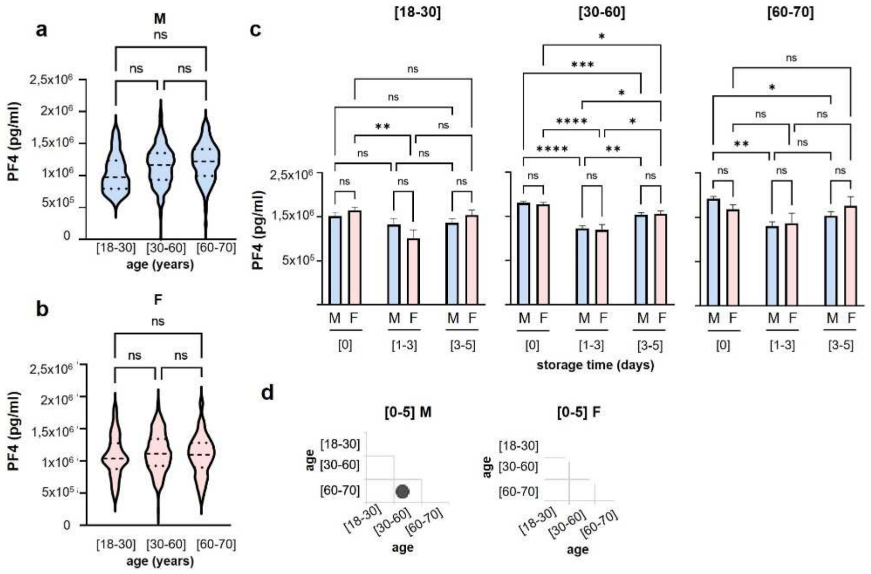


**Supplemental Figure 2. Evaluation of SDA-PC PF4 levels depending on donor sex.** Volocity plot of the expression of SDA-PC PF4 from male (a) donor (M) or female (b) donor (F) based on different donor age, 2-way ANOVA, non-significative difference (ns) ; large dash present median of data and small dash the quartiles. (c) PF4 expression during storage period regardless of donor age, 2-way ANOVA, *$p<0.05$**$p<0.01$*** $p < 0.001$ **** $p < 0.0001$. Light Blue for male donor (M) and light pink for female donor (F). (d) Spearman's correlation matrix between PF4

concentration and sex/age of the donor. White square corresponds to no correlation with p value < 0.05. Dark blue dot corresponds to negative correlation with p-value <0.05.
